# The Mitochondrial Genome of the Entomoparasitic Green Alga *Helicosporidium*

**DOI:** 10.1371/journal.pone.0008954

**Published:** 2010-01-29

**Authors:** Jean-François Pombert, Patrick J. Keeling

**Affiliations:** Department of Botany, University of British Columbia, Vancouver, British Columbia, Canada; University of California, Riverside, United States of America

## Abstract

**Background:**

Helicosporidia are achlorophyllous, non-photosynthetic protists that are obligate parasites of invertebrates. Highly specialized, these pathogens feature an unusual cyst stage that dehisces inside the infected organism and releases a filamentous cell displaying surface projections, which will penetrate the host gut wall and eventually reproduce in the hemolymph. Long classified as *incertae sedis* or as relatives of other parasites such as Apicomplexa or Microsporidia, the Helicosporidia were surprisingly identified through molecular phylogeny as belonging to the Chlorophyta, a phylum of green algae. Most phylogenetic analyses involving Helicosporidia have placed them within the subgroup Trebouxiophyceae and further suggested a close affiliation between the Helicosporidia and the genus *Prototheca*. *Prototheca* species are also achlorophyllous and pathogenic, but they infect vertebrate hosts, inducing protothecosis in humans. The complete plastid genome of an *Helicosporidium* species was recently described and is a model of compaction and reduction. Here we describe the complete mitochondrial genome sequence of the same strain, *Helicosporidium* sp. ATCC 50920 isolated from the black fly *Simulium jonesi*.

**Methodology/Principal Findings:**

The circular mapping 49343 bp mitochondrial genome of *Helicosporidium* closely resembles that of the vertebrate parasite *Prototheca wickerhamii*. The two genomes share an almost identical gene complement and display a level of synteny that is higher than any other sequenced chlorophyte mitochondrial DNAs. Interestingly, the *Helicosporidium* mtDNA feature a trans-spliced group I intron, and a second group I intron that contains two open reading frames that appear to be degenerate maturase/endonuclease genes, both rare characteristics for this type of intron.

**Conclusions/Significance:**

The architecture, genome content, and phylogeny of the *Helicosporidium* mitochondrial genome are all congruent with its close relationship to *Prototheca* within the Trebouxiophyceae. The *Helicosporidium* mitochondrial genome does, however, contain a number of novel features, particularly relating to its introns.

## Introduction

Helicosporidia are single cell parasitic eukaryotes infecting a wide range of insects ([1] and references therein). These entomopathogens feature three different life stages: cysts, filamentous cells and vegetative cells. When the infectous cysts burst open within the gut of their host, they release a filamentous cell with surface barbs along with three egg-shaped accessory cells [2]. The barbed filaments proceed to invade the gut cells, passing though them and emerging into the hemolymph [3]. In their vegetative state within the hemolymph, Helicosporidia reproduce by several rounds of autosporogenic division within the pellicle of the mother cell, with each autosporulation producing up to eight daugther cells [4]. Generally, the infection leads to the death of the host, but the exact mode of transmission remains poorly known.

First described in 1921 by Keilin [5], the Helicosporidia were long ignored in classification systems due to their mysterious origins ([3], [6] and references therein). Initially, they were ascribed to the Protozoa, then transferred to the Fungi, before being reclassified as Protozoa, specifically within the Cnidosporidia. Cnidosporidia were a longstanding group consisting of Helicosporidia, Microsporidia, and Myxosporidia. The latter two groups are now known to be fungi and animals, respectively, so it is fitting that the Helicosporidia should eventually be determined to be closely related to plants, or more specifically to green algae. This was first suggested based on the astute observation that the morphology and *in vitro* development of Helicosporidia resemble that of the achlorophyllous non-photosynthetic green algae of the genus *Prototheca* [3]. This taxonomic affiliation was quickly supported by molecular phylogeny of several nucleus-encoded genes [6], [7], [8], [9], and further supported by the subsequent finding that Helicosporidia harbour a functional yet heavily reduced chloroplast genome [10], [11].

Phylogenetic analyses have, where the sampling diversity was sufficient, consistently suggested an affiliation to *Prototheca* [6], [7], [8], [9], [12], in agreement with their morphological characters [3]. *Prototheca* is a member of the green algal class Trebouxiophyceae, and is also achlorophyllous and pathogenic. This association is intriguing since protothecans infect only vertebrates inducing protothecosis in humans [13], whereas the Helicosporidia are known so far to invade only invertebrates.

To learn more about these intriguing but poorly studied parasites, and further compare them with their likely closest relatives in the genus *Prototheca*, here we report the complete sequence of the *Helicosporidium* sp. ATCC 50920 mitochondrial genome. The mitochondrial genome is a useful tool for such comparisons because complete mitochondrial genomes are available from representatives of most major groups of green algae, including *Prototheca*. The architecture of the 49343 pb-long *Helicosporidium* mitochondrial genome and the 60 genes it encodes are highly similar to those of *Prototheca wickerhamii* and display a level of synteny that have not been previously observed between any two chlorophyte mitochondrial DNAs (mtDNAs). The *Helicosporidium* mtDNA also has several interesting characteristics that are not only absent from *Prototheca*, but are rare in mitochondria as a whole, including a rare case of group I intron spliced in *trans* and introns that encode multiple ORFs.

## Results

### Main Features of the Mitochondrial Genome

The *Helicosporidium* mitochodondrial genome (GenBank: GQ339576) was sequenced as part of an ongoing genome project on *Helicosporidium* sp. strain ATCC 50920 in which 402658 reads totalizing 146.7 Mbp were generated by 454 Titanium pyrosequencing. Over 87.5% of these reads (364 bp average) were assembled into 4360 contigs representing about 10.6 Mbp. The mitochondrial genome was represented by a single contig comprising 53785 reads, amounting to 1.96 Mbp, or 396-fold coverage of the genome

**Figure 1:**
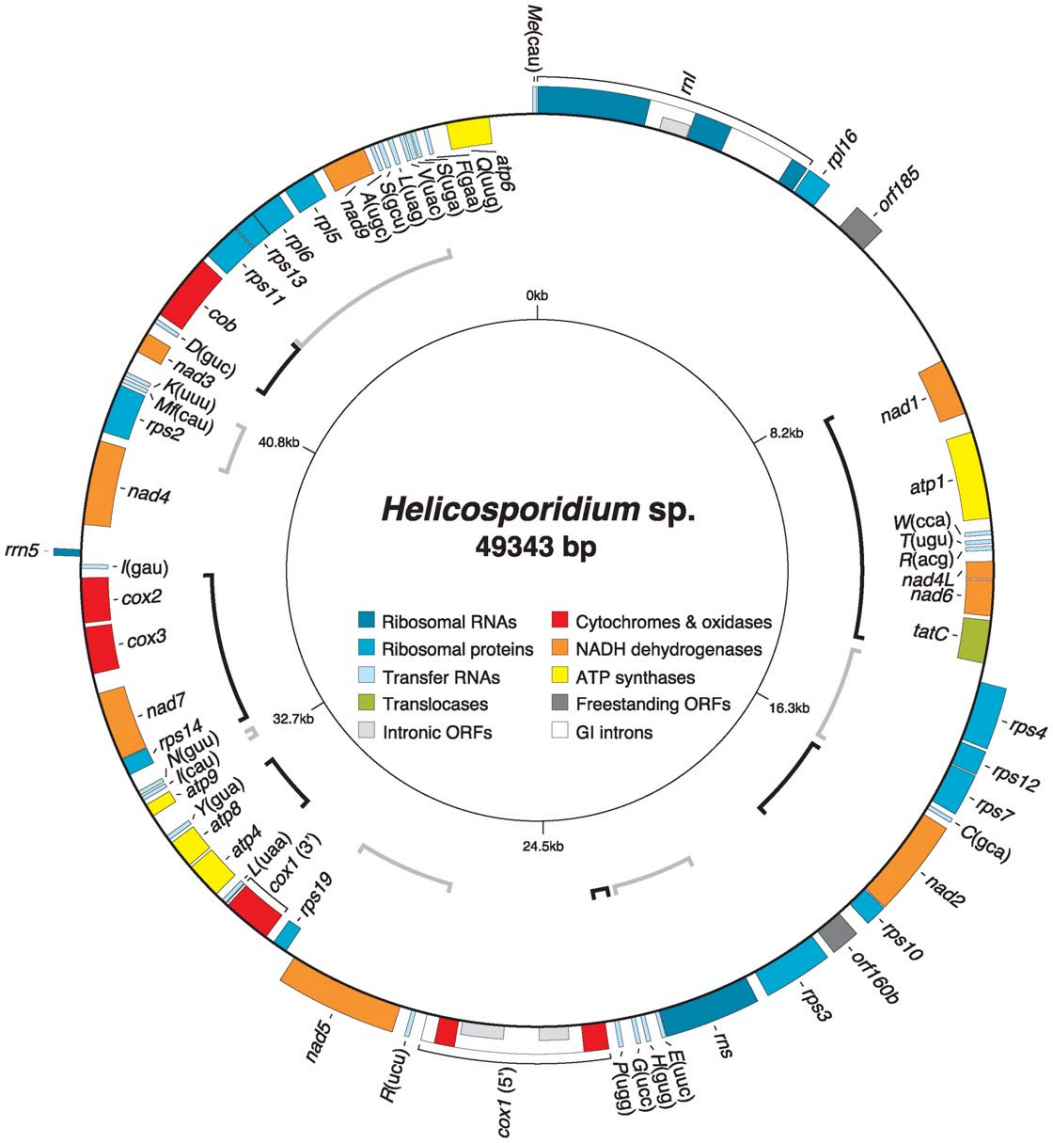
Gene map of *Helicosporidium* mtDNA. Genes (filled boxes) located outside/inside the map are transcribed clockwise/counterclockwise. Introns are denoted by open boxes whereas intronic ORFs are illustrated as half-height boxes within the open boxes. tRNA genes are indicated by the one-letter amino acid code followed by the anticodon in parentheses (Me, elongator methionine; Mf, initiator methionine). ORFs smaller than 150 amino acids are not shown. Shared clusters between the *Helicosporidium* and *Prototheca* mitochondrial genomes are denoted by alterning black and gray brackets.

The mitochondrial genome maps as a circular molecule of 49343 bp (Figure 1) featuring an overall A+T content of 74.4% (Table 1). The 60 genes it encodes are distributed with a marked strand polarity, but are not as symmetrical as those of *Prototheca*. The *Helicosporidium* mtDNA contains a total of four introns, all group I, which split the *rnl* and *cox1* genes in three exons each. The *Helicosporidium* mtDNA also features three intronic open reading frames (ORFs) and two freestanding ORFs that are longer than 150 codons. Intergenic regions in the *Helicosporidium* mitochondrial genome range from 0 to 2355 bp, with an average of 183 bp, and no overlapping genes. The *Helicosporidium* mtDNA is more densely packed than that of *Prototheca* and is leaner by about 6 kbp despite maintaining a near-identical gene complement, differing only by a single tRNA, *trnG*(gcc) (Tables S1 and S2). Both genomes features *trnT*(ugu), a tRNA-encoding gene also found within the mtDNA of the ulvophycean alga *Pseudendoclonium* (Table S2). Like *Prototheca* and *Pseudendoclonium* mtDNAs, the *Helicosporidium* mitochondrial genome harbors a self-sufficient tRNA gene complement able to decode all codons assuming super Wobble codon/anticodon interactions. Codon usage in *Helicosporidium* mtDNA (Table S3) is also similar to that of *Prototheca* mtDNA, which parallels their very similar A+T content.

**Table 1:** Main features of *Helicosporidium* and other chlorophyte mtDNAs.

Chlorophyte mtDNA	Size (bp)	A+T content (%)	Gene content^a^	Gene density^b^	Coding seq. (%)^c^	Introns (I/II)	Intron ORFs (I/II)
Prasinophyceae							
*Nephroselmis*	45223	67.2	65	1/696	80.6	4/0	4/0
*Ostreococcus*	44237	61.8	65	1/590	92.1	0/0	0/0
Trebouxiophyceae							
*Helicosporidium*	49343	74.4	60	1/822	75.9	4/0	3/0
*Prototheca*	55328	74.2	61	1/907	70.6	5/0	2/0
Ulvophyceae							
*Oltmannsiellopsis*	56761	66.6	54	1/1051	68.7	2/1	2/1
*Pseudendoclonium*	95880	60.7	57	1/1682	58.7	7/0	6/0
Chlorophyceae							
*C. eugametos*	22897	65.4	12	1/1908	84.6	9/0	7/0
*C. reinhardtii*	15758	54.8	12	1/1313	83.1	0/0	0/0
*Chlorogonium*	22704	62.2	12	1/1892	89.1	6/0	6/0
*Scenedesmus*	42919	63.7	42	1/1022	60.6	2/2	1/0
Uncertain affiliation							
*Pedinomonas*	25137	77.8	22	1/1143	60.5	0/1	0/0

a Duplicated genes, unique ORFs and intron ORFs were not taken into account.

b Duplicated genes were taken into account (size/number of genes).

c Conserved genes (unique and duplicated), ORFs, introns and introns ORFs were considered as coding sequences.

The two freestanding ORFs in *Helicosporidium* mtDNA (*orf160b* and *orf185*) showed no significant homology in BLAST searches (*E*-values ≤1E-05). Although *orf160b* shares no identifiable similarity to any known ORF, it is located at the same genetic locus as *ymf45* (*orf174*) in *Prototheca* mtDNA, *i.e.* between the *nad2*-*rps10* and *rps3* genes. Given their small size, the two *Helicosporidium* mtDNA freestanding ORFs might not encode any relevant biological product and rather represent random open reading frames, but the conserved position of *Helicosporidium orf160b* and *Prototheca orf174* does suggest they are rapidly diverging homologues.

### Synteny

The *Helicosporidium* mitochondrial genome features a high level of synteny with that of *Prototheca*. The two genomes share 12 gene clusters encompassing a total of 45 genes (Figure 1). This level of synteny has not been previously observed between mitochondrial genomes of any other chlorophytes. The mitochondrial genomes of the prasinophytes *Nephroselmis* and *Ostreococcus* share 10 clusters comprising a total of 36 genes, those of the ulvophytes *Pseudendoclonium* and *Oltmannsiellopsis* share only two gene pairs (4 genes) despite displaying a similar gene complement [14], whereas in the Chlorophyceae the two more similar mtDNAs (*Chlorogonium* and *C. eugametos*) share 3 clusters (8 genes). The *Helicosporidium* mtDNA shares six clusters (13 genes) and five clusters (11 genes) with the prasinophycean mtDNAs of *Ostreococcus* and *Nephroselmis,* respectively, and none with the ulvophycean or chlorophycean mitochondrial genomes.

Given the level of synteny between the *Helicosporidium* and *Prototheca* mitochondrial genomes, a minimum of 24 permutations by inversion between the 60 genes they share would be sufficient to convert the structure of one genome into that of the other. In contrast, at least 30 permutations (63 genes shared) and 46 permutations (50 genes shared) would be required to interconvert the structure of the *Nephroselmis* and *Ostreococcus* mtDNAs and of the *Pseudendoclonium* and *Oltmannsiellopsis* mtDNAs, respectively. However this number does not account for the creation of the inverted repeats in *Ostreococcus* due to the limitations of the GRIMM algorithm and is therefore an underestimate. In the Chlorophyceae, the gene-poor mtDNAs of *Chlorogonium* and *C. eugametos* are more closely related to each other (12 genes shared, 7 permutations) than to that of *C. reinhardtii* (12 genes shared, 19 and 18 permutations, respectively). The 42-gene mtDNA of *Scenedesmus* was not compared to the other gene-poor mtDNAs from the Chlorophyceae.

### Introns

**Figure 2:**
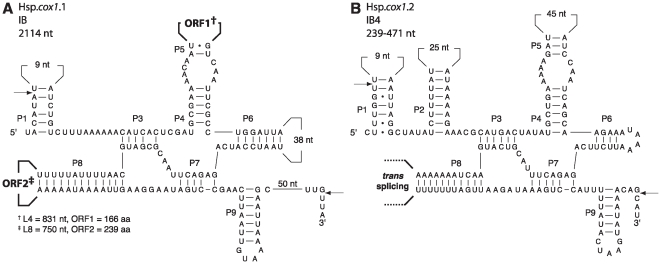
Predicted secondary structures of *Helicosporidium cox1* group I introns. The group I introns displayed according to Burke *et al* [36] were classified according to Michel and Westhof [37]. The Hsp.*cox1*.1 intron could not be assigned unambiguously to a subgroup of IB introns. Splice sites between exon and intron residues are denoted by arrows. Canonical Watson-Crick base pairings are denoted by dashes whereas guanine-uracyl pairings are marked by dots. Numbers inside variable loops indicate the sizes of these loops. The size of the L8 loop in the Hsp.*cox1*.2 intron is uncertain; the junction between the two parts of this trans-spliced intron occurs in the L8 loop. The putative LAGLIDADG endonucleases encoded within the intronic ORFs each contain a single copy of this motif.

The *Helicosporidium* mitochondrial genome contains a total of four group I introns inserted into the *cox1* and *rnl* genes (Figures 2 and S1). Although *Prototheca* mtDNA also has five introns within these two genes, none are located at cognate sites within *Helicosporidium* mtDNA. Interestingly, the Hsp.*cox1*.1 intron (Figure 2A) contains two distinct open reading frames, *orf166* and *orf239*, located in different variable loops (L4 and L8, respectively). Both ORFs display a single dodecapeptide LAGLIDADG motif, indicative of a putative endonuclease function for these proteins. However, functional LAGLIDADG endonucleases contain two dodecapeptide motifs [15], raising the interesting possibility that the two ORFs generate an heterodimer constituted of one product in the N-terminal domain and of the other product in the C-terminal portion of the endonuclease. Alternatively, the two ORFs may also code for two independent homodimeric endonucleases. Homodimeric LAGLIDADG endonucleases are commonly found in group I introns, although the presence of two endonucleases in a single intron is extremely rare.

**Figure 3:**
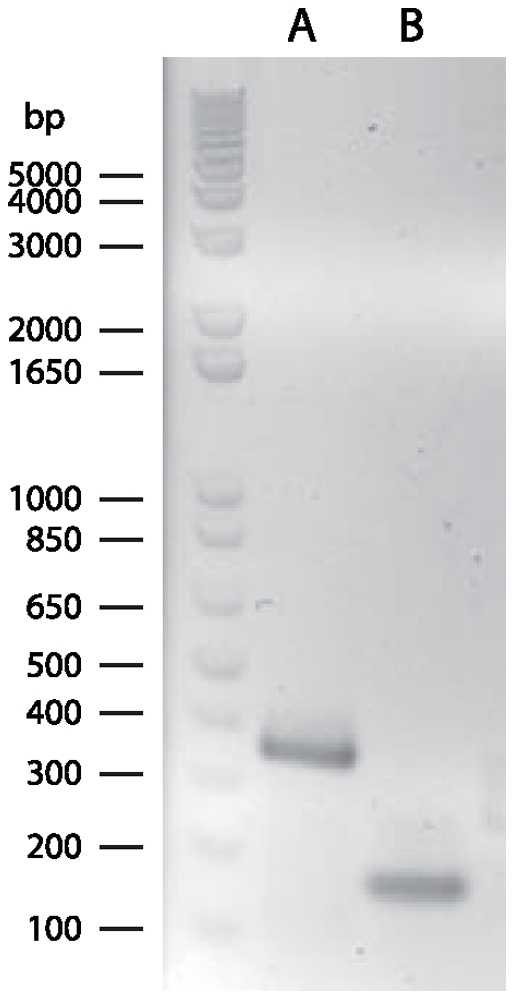
Electrophoretic analysis of RT-PCRs performed on *Helicosporidium* total RNA. The amplicon in lane A corresponds to the expected size (343 bp) between the two internal *cox1* primers in exons 2 and 3 (Hecox1F & Hecox1R) after *trans*-splicing of the Hsp.*cox1*.2 group I intron. The amplicon in lane B corresponds to the *in cis* positive control (166 bp) performed with internal *atp1* primers (07487R & 00085R).

Perhaps the most surprising finding is that the *Helicosporidium* mtDNA contains a *trans*-spliced group I intron. The Hsp.*cox1*.2 intron (Figure 2B) is fragmented into two pieces that are located on different strands and separated by three genes (*trnR*(ucu), *nad5*, *rps19*) spanning over 3 kb. This fragmentation is a genuine feature of the genome, supported by an assembly in which the mean coverage is about 400X. Because the *cox1* gene is conserved among all chlorophyte mtDNAs and because its product, the first subunit of the mitochondrial cytochrome oxidase, is likely essential to the mitochondrion, we expected the *trans*-splicing of this intron to occur at the mRNA level so that a functional protein could be produced. To test this, we performed RT-PCRs on *Helicosporidium* RNA treated with DNAse using primers specific for the second and third *cox1* exons. As expected, we obtained a 343 bp fragment consisting of the spliced exons (Figure 3). Sequencing of the 343 bp amplicon confirmed the 5′ and 3′ exon/intron splice junctions of the Hsp.*cox1*.2 intron illustrated in Figure 2B. When DNA was used as template for PCRs with the same primers, no amplicon was observed.

To our knowledge, *trans*-spliced group I introns were never reported until this year, and presently only three other instances are known: all in the mitochondrial *cox1* gene. The first of these is in the mtDNA of the lycophyte *Isoetes engelmannii* [16] while the other two were identified by reinvestigation of the *Trichoplax adhaerens* mitochondrial genome [17]. However, the Hsp.*cox1*.2 intron is not inserted at a cognate site with any of these *trans*-spliced introns (Figure S2). Also, aside from its canonical group I intron structure, the Hsp.*cox1*.2 intron does not display strong identity with the *Isoetes* and *Trichoplax* introns even though it does share the same L8 *trans*-splicing location as the two *Trichoplax* introns.

### Repeated Elements

The *Helicosporidium* mtDNA is not rich in repeated elements. Most of the repeated nucleotide strings detectable in the *Helicosporidium* mitochondrial genome are confined to AT-rich intergenic regions, are short, and are composed of adenosine and/or thymine residues arranged either in stretches or as alternating bases. The distribution of repeated elements oberved in *Helicosporidium* mtDNA (Figure S3) parallels that of *Prototheca* mtDNA in which the presence of A+T-rich repeats arrayed in tandem has been previously reported [18]. As in *Prototheca* mtDNA, the repeated elements are dispersed throughout the whole genome sequence in intergenic regions and in introns.

### Phylogeny

**Figure 4:**
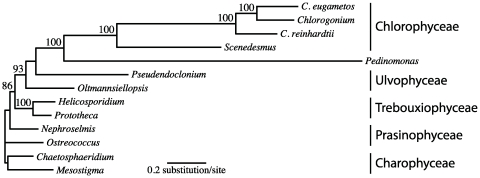
Phylogenetic position of *Helicosporidium* sp. ATCC 50920 as inferred from amino acid sequences derived from the seven protein-encoding genes that are shared between all sequenced chlorophyte mtDNAs. The best ML-tree inferred with PHYML 3.0 under the LG+Γ4+F+I model of amino acid substitutions is shown here (13 taxa, 2362 positions, 1373 phylogenetically informative). The charophyte green algae *Mesostigma viride* and *Chaetosphaeridium globosum* were used as outgroups. Bootstrap values over 80% are shown above the corresponding nodes. Branch lenghts are drawn to scale. *Pedinomonas minor* has not yet been assigned unambiguously to one the four classes of chlorophyte green algae.

The availability of the *Helicosporidium* mitochondrial genome provides us with another opportunity to probe the phylogenetic position of the Helicosporidia, and a useful opportunity because all major subgroups of green algae are available for analysis (this is not true for many nuclear genes, and the plastid genome does not contribute substantially to the question since the *Prototheca* plastid genome has not been sequenced and that of *Helicosporidium* is so reduced as to be difficult to compare with its photosynthetic relatives). As expected from and congruent with previous phylogenetic analyses [3], [6], [7], [8], [9], [12], phylogenies inferred from amino acid sequences derived from the seven protein-encoding genes that are shared between all mitochondrial genomes of chlorophytes supported a close affiliation between *Helicosporidium* and *Prototheca*. The two pathogenic achlorophyllous algae were joined together in all analyses (Figure 4). This affiliation was not dependent on the method of phylogenetic reconstruction, and was recovered in ML, Bayesian and even MP analyses. Given the overall level of support for the *Helicosporidium*/*Prototheca* affiliation, the placement of the helicosporidian parasites within the Trebouxiophyceae is most likely genuine.

## Discussion

The mitochondrial genome of the obligately parasitic green alga *Helicosporidium* stands out in two different ways. First, it strongly supports the relationship between *Helicosporidium* and *Prototheca*, but not just because it provides a large molecular data set from which phylogenies can be inferred, but also because of their shared genomic structure. Based on the low levels of gene order conservation in other green algal mitochondrial genomes, we might expect to see few blocks of conservation between *Helicosporidium* and *Prototheca*. Clearly this was not the case for *Helicosporidium* and *Prototheca*, because their mtDNAs display a surprisingly high level of similarity in form. This close resemblance is probably best explained by a recent split between these two species. The only chlorophyte mtDNAs that display a comparable level of similarity are those of the prasinophytes *Nephroselmis* and *Ostreococcus*, but even here the level of conservation is much lower, with one featuring an inverted repeat that is missing from the other. In the Chlorophyceae, the gene-poor mtDNAs of *Chlorogonium* and *C. eugametos* also display an appreciable level of synteny, with 8 of their 12 genes (66%) being located in shared clusters, although this percentage is still lower than that observed between *Helicosporidium* and *Prototheca* mtDNAs (75%), and there are far fewer combinations of 12 genes than of 60.

A second standout feature of the *Helicosporidium* mtDNA is in its introns, and in particular the presence of a group I intron that splices in *trans*. Although *trans*-splicing in various group II introns has been known to occur for some time (reviewed in [19], [20]), the first examples of *trans*-spliced group I introns have only been described recently [16], [17]. Like other known *trans*-spliced group I introns, the predicted secondary structure of the Hsp.*cox1*.2 intron (Figure 2) closely conforms to a canonical group I intron, suggesting it most likely arose from a *cis*-spliced group I intron that was broken into two pieces, but that could still fold at the mRNA level to produce a functional ribozyme. Because this fragmentation occurred within a variable loop, its effect on the intron self-splicing capability may have been minimal (although presumably if such an event had little impact it would occur more frequently than it does). It is unclear what effect such a fragmentation might have on the viability of the intron if it occurred in core regions like the P7 pairings. However, as ribozymes derived from group I introns can catalyse *trans*-excision-splicing reactions in other RNA molecules [21], [22], [23], their functional core may be somewhat malleable.

The four *trans*-spliced group I introns known so far most likely arose independently. Not only do they appear dissimilar at the nucleotide level outside of their canonical group I intron structure, but despite their shared location within the mitochondrial *cox1* gene, none are inserted at cognate sites. Also to be transferred horizontally from one organism to another, at least two recombinational events would be required, one for each *trans*-spliced segment. This appears unlikely, especially considering that such recombinational events would likely involve the adjacent exons. As *Helicosporidium*, *Trichoplax* and *Isoetes* are evolutionary distant and belong to very different lineages, recombination between their genes, even as conserved as *cox1*, is not a very compelling hypothesis. It is perhaps not surprising that these rare introns were first discovered within the *cox1* gene, given its conservation and its importance for the mitochondrion. Other existing instances of *trans*-splicing group I introns in less conserved genes may have been overlooked, and reinvestigation of sequenced mitochondrial genomes, as performed by Burger and coauthors on the *Trichoplax* mtDNA [17], may reveal more of these segmented yet functional ribozymes.

The homing endonucleases encoded within intronic ORFs confer mobility to the intron host by permitting double strand breaks of a target DNA. Very often, these endonucleases are lost and the introns lacking these ORFs are no longer considered mobile. It is very rare however to find an intron containing two distinct ORFs coding for putative endonucleases. Given that different homing endonucleases usually have different DNA targets, the presence of two such proteins tentatively confers a greater protential for mobility and self-propagation of the intron. It is unknown if the two ORFs present in the *Helicosporidium* Hsp.*cox1*.1 intron code for functional and expressed endonucleases. If so, it would be interesting to determine whether they act separately or as a heterologous unit.

### Conclusions

The structure and content of the *Helicosporidium* mitochondrial genome, as well as the phylogenetic inferences derived from the sequences it encodes, support the specific relationship to the genus *Prototheca*. The introns of this genome also have a number of interesting characteristics rarely seen in other organelle genomes. Our results, combined with the previously published plastid genome sequence of *Helicosporidium* sp. ATCC 50920 [21], complete the deciphering of this peculiar species's organellar genetic imprint. The sequencing of the *Helicosporidium* sp. ATCC 50920 nuclear genome would provide us with a global picture that, hopefully, would yield clues into the adaption of this alga from a free-living entity to that of an entomoparasite. Also, a comparative approach with its protothecan relatives would give us interesting insights into the nature of their selective parasitism.

## Materials and Methods

### PCRs and RT-PCRs

PCRs were performed using 22- to 24-mers and the EconoTaq PLUS GREEN kit from Lucigen (Middleton, WI, USA) with 35 cycles of denaturation (1 min at 94°C), annealing (1 min at 55°C) and elongation (3 min at 72°C). RT-PCRs were performed using the SuperScript One-Step RT-PCR with Platinum *Taq* kit from Invitrogen (Carlsbad, CA, USA) with an initial cDNA synthesis cycle (30 min at 50°C) followed by a 2 min denaturation cycle at 94°C. A total of 35 amplification cycles of denaturation (15 sec at 94°C), annealing (30 sec at 55°C), and elongation (1 min at 70°C) were then performed. The Hecox1F (5′-CTCTTCCTGTATTAGCTGGTGG-3′) and Hecox1R (5′-GCAATAATCATTGTAGCTGCAG-3′) and the 07487R (5′-CAATTGTAGACGTACCAGTTGG-3′) and 00085R (5′-GTTTGCATAGGTTGGCTTACAG-3′) primers were used in RT-PCRs.

### Genome Sequencing

The *Helicosporidium* mtDNA was sequenced using the massively parallel GS-FLX DNA pyrosquencing platform from Roche 454 Life Sciences (Branford, CT, USA).

The creation of the *Helicosporidium* mtDNA GS-FLX shotgun library and the GS-FLX 454 pyrosequencing (using the GS-FLX Titanium reagents) were carried out by the McGill University and Génome Québec Innovation Centre. The Newbler assemblies obtained from Génome Québec were converted to, edited, and assembled with CONSED 19 [24]. Ambiguous regions in the assemblies were either (1) edited according to their conceptual translations or (2) amplified by PCR with 22-mers primers flanking the ambiguous regions, sequenced using traditional Sanger chemistry by Macrogen (Seoul, Korea) and then edited according to Sanger base calling.

### Genome Annotation and Analysis

Genes were identified by Blast homology searches [25] against a local copy of the National Center for Biotechnology Information (NCBI) nonredundant database using the NCBI BLASTALL suite (http://www.ncbi.nlm.nih.gov/Ftp/blast). Positions of open reading frames and protein-coding genes were determined using GETORF from EMBOSS 6.0.1 [26] and ORFFINDER at NCBI, whereas positions of tRNA-encoding genes were determined with tRNAscan-SE [27]. Insertions sites of group I introns and their predicted secondary structures were determined manually. Codon usage in protein-encoding genes was determined with CUSP from the EMBOSS package. Repeated elements were first visualized with PipMaker [28]. Then, repeated elements arrayed in tandem were identified with ETANDEM from the EMBOSS package whereas dispersed repeated elements were located with REPuter 2.74 [29]. Potential hairpin structures were screened for with PALINDROME from the EMBOSS package. Minimal number of permutations by inversions between mitochondrial genomes were inferred with GRIMM [30]. For this analysis, the *trans*-spliced exons of the *cox1* gene in *Helicosporidium* mtDNA and the fragmented rRNA genes in chlorophycean mtDNAs were coded as distinct fragments. Also, as GRIMM cannot handle duplicate genes, one copy of the *Ostreococcus* inverted repeats was removed.

### Phylogenetic Analyses

In addition to the *Helicosporidium* mitochondrial genome sequenced by the authors [GenBank:GQ339576], the following mtDNAs used in this study were retrieved from GenBank: *Chaetosphaeridium globosum* [GenBank:NC_004118], *Chlamydomonas eugametos* [GenBank:NC_001872], *Chlamydomonas reinhardtii* [GenBank:NC_001638], *Chlorogonium elongatum* [Genbank:Y13643, Y13644, Y07814], *Mesostigma viride* [GenBank:NC_008240], *Nephroselmis olivacea* [GenBank:NC_008239], *Oltmannsiellopsis viridis* [GenBank:NC_008256], *Ostreococcus tauri* [GenBank:NC_008290], *Pedinomonas minor* [GenBank:NC_000892], *Prototheca wickerhamii* [GenBank:NC_001613], *Pseudendoclonium akinetum* [GenBank:NC_005926], *Scenedesmus obliquus* [GenBank:NC_002254]. Mitochondrial protein sequences were inferred from the conceptual translation of the seven protein-encoding genes that are share between all chlorophyte mtDNAs. The amino acid sequences were aligned using T-COFFEE 7.81 [31], the ambiguous regions within these alignments filtered with GBLOCKS 0.91 b [32], and the filtered individual sequences concatenated. Maximum Likelihood computations were performed using PHYML 3.0 [33] under the LG+Γ4+F+I model of amino acid substitution selected with ProtTest 2.0 [34]. Bayesian inferences were performed with PhyloBayes 3.2 [35] under the CAT+ Γ4 model of amino acid substitution running two concurrent chains terminated using PhyloBayes automatic stopping rule (maxdiff <0.3).

## Supporting Information

Figure S1 Predicted secondary structures of *Helicosporidium rnl* group I introns. The *rnl* group I introns displayed according to Burke *et al* were classified according to Michel and Westhof. Splice sites between exon and intron residues are denoted by arrows. Canonical Watson-Crick base pairings are denoted by dashes whereas guanine-uracyl pairings are marked by dots. Numbers inside variable loops indicate the sizes of these loops. The putative LAGLIDADG endonuclease encoded within the Hsp.*rnl*.1 intronic ORF contains a single copy of this motif.
(0.91 MB EPS)

Figure S2 *Trans*-spliced GI introns insertion sites. The insertion sites of the *Helicosporidium*, *Isoetes* and *Trichoplax trans*-spliced group I introns are indicated by arrows on the Cox1 amino acid alignment (positions 225 to 464 shown). Numbers on the right of the alignment indicate the amino acid positions on the corresponding sequences. The different trans-spliced introns are indicated by roman numerals: I, *Helicosporidium* (Hsp.*cox1*.2); II, *Trichoplax* (*cox1* intron 4); III, *Trichoplax* (*cox1* intron 5); IV, *Isoetes* (coxi1305). The *Trichoplax* intron insertion sites were taken from Burger *et al*.
(4.97 MB EPS)

Figure S3 Densities of repeated elements in *Helicosporidium* and other chlorophyte mitochondrial genomes. Repeated elements identified with REPuter are connected by lines on the corresponding mtDNA circular representations (adapted from Kurtz). Repeats of at least 15, 30 and 45 nt are shown on the left, middle and right panels respectively. For this analysis, one copy of the *Ostreococcus* mtDNA inverted repeats has been removed. Also, the linear *C. reinhardtii* mitochondrial genome is represented here as a circle.
(2.22 MB PDF)

Table S1 Gene repertoires of *Helicosporidium* and other chlorophyte mtDNAs. ^a^ Nol, *Nephroselmis olivacea*; Ota, *Ostreococcus tauri*; Hsp, *Helicosporidium* sp.; Pwi, *Prototheca wickerhamii*; Ovi, *Oltmannsiellopsis viridis*; Pak, *Pseudendoclonium akinetum*; Sob, *Scenedesmus obliquus*; Pmi, *Pedinomonas minor*; Cre, *Chlamydomonas reinhardtii*; Ceu, *Chlamydomonas eugametos*; Cel, *Chlorogonium elongatum*. Presence/absence of a gene is denoted by +/−. ^b^ Gene fragmented in corresponding mtDNA.
(0.10 MB DOC)

Table S2 tRNA gene repertoires of *Helicosporidium* and other chlorophyte mtDNAs. ^a^ Nol, *Nephroselmis olivacea*; Ota, *Ostreococcus tauri*; Hsp, *Helicosporidium* sp.; Pwi, *Prototheca wickerhamii*; Ovi, *Oltmannsiellopsis viridis*; Pak, *Pseudendoclonium akinetum*; Sob, *Scenedesmus obliquus*; Pmi, *Pedinomonas minor*; Cre, *Chlamydomonas reinhardtii*; Ceu, *Chlamydomonas eugametos*; Cel, *Chlorogonium elongatum*. Presence/absence of a gene is denoted by +/−. ^b^ Me, elongator methionine; Mf, initiator methionine. ^c^ Genome specifies a single *trnM*(cau).
(0.09 MB DOC)

Table S3 Codon usage in the 32 protein-encoding genes of *Helicosporidium* mtDNA. ^a^ Percentage of each amino acid specified by the specified codon. ^b^ Anticodon of *Helicosporidium* mtDNA-encoded tRNA recognizing the corresponding codon. The following tRNAs with an uracyl in the first position of their anticodon are assumed to decode all four members of the four-codon families: alanine, GCN; glycine, GGN; leucine, CUN; proline, CCN; serine, UCN; threonine, ACN; valine, GUN. ^c^ Amino acids are labelled by their one-letter IUPAC code. Termination codons are indicated by asterisks. ^d^ In chlorophytes mtDNAs, the gene coding for tRNA^Thr^ (ugu) has been found only within the mitochondrial genomes of *Helicosporidium*, *Prototheca* and *Pseudendoclonium*. ^e^ The initiator and elongator tRNA^Met^ (cau) are encoded by different genes.
(0.07 MB DOC)
