# Supplementary figures and images for: The Mitochondrial Genome of the Entomoparasitic Green Alga Helicosporidium
Source: PLoS One. 2010 Jan 29;5(1):e8954. doi: 10.1371/journal.pone.0008954 (PMC2813288; doi:10.1371/journal.pone.0008954)

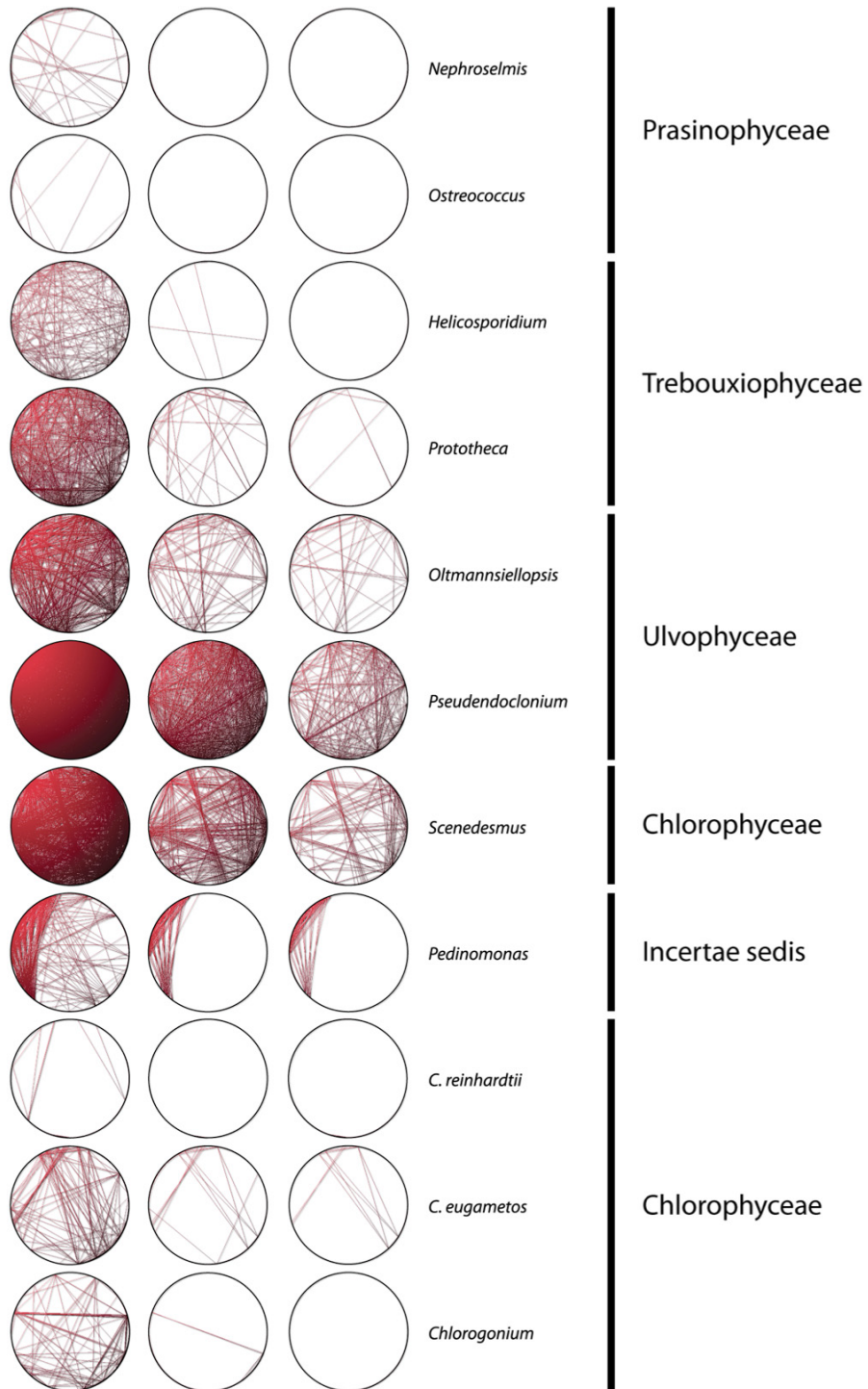

Supplement: Figure S3 — Densities of repeated elements in Helicosporidium and other chlorophyte mitochondrial genomes. Repeated elements identified with REPuter are connected by lines on the corresponding mtDNA circular representations (adapted from Kurtz). Repeats of at least 15, 30 and 45 nt are shown on the left, middle and right panels respectively. For this analysis, one copy of the Ostreococcus mtDNA inverted repeats has been removed. Also, the linear C. reinhardtii mitochondrial genome is represented here as a circle. (2.22 MB PDF) [file pone.0008954.s003.pdf]
