# Supplementary material for: The Mitochondrial Genome of the Entomoparasitic Green Alga Helicosporidium
Source: PLoS One. 2010 Jan 29;5(1):e8954. doi: 10.1371/journal.pone.0008954 (PMC2813288; doi:10.1371/journal.pone.0008954)
Supplement: Table S1 — Gene repertoires of Helicosporidium and other chlorophyte mtDNAs. a Nol, Nephroselmis olivacea; Ota, Ostreococcus tauri; Hsp, Helicosporidium sp.; Pwi, Prototheca wickerhamii; Ovi, Oltmannsiellopsis viridis; Pak, Pseudendoclonium akinetum; Sob, Scenedesmus obliquus; Pmi, Pedinomonas minor; Cre, Chlamydomonas reinhardtii; Ceu, Chlamydomonas eugametos; Cel, Chlorogonium elongatum. Presence/absence of a gene is denoted by +/−. b Gene fragmented in corresponding mtDNA. (0.10 MB DOC) [file pone.0008954.s004.doc]

| Gene | Nol^a^ | Ota | Hsp | Pwi | Ovi | Pak | Sob | Pmi | Cre | Ceu | Cel |
| --- | --- | --- | --- | --- | --- | --- | --- | --- | --- | --- | --- |
| Complex I proteins | | | | | | | | | | | |
| *nad1* | + | + | + | + | + | + | + | + | + | + | + |
| *nad2* | + | + | + | + | + | + | + | + | + | + | + |
| *nad3* | + | + | + | + | + | + | + | + | - | - | - |
| *nad4* | + | + | + | + | + | + | + | + | + | + | + |
| *nad4L* | + | + | + | + | + | + | + | + | - | - | - |
| *nad5* | + | + | + | + | + | + | + | + | + | + | + |
| *nad6* | + | + | + | + | + | + | + | + | + | + | + |
| *nad7* | + | + | + | + | + | + | - | - | - | - | - |
| *nad9* | + | + | + | + | + | - | - | - | - | - | - |
| *nad10* | + | + | - | - | - | - | - | - | - | - | - |
| Complex III proteins | | | | | | | | | | | |
| *cob* | + | + | + | + | + | + | + | + | + | + | + |
| Complex IV proteins | | | | | | | | | | | |
| *cox1* | + | + | + | + | + | + | + | + | + | + | + |
| *cox2* | + | + | + | + | + | + | + | - | - | - | - |
| *cox3* | + | + | + | + | + | + | + | - | - | - | - |
| Complex V proteins | | | | | | | | | | | |
| *atp1* | + | + | + | + | + | + | - | - | - | - | - |
| *atp4* | + | + | + | + | + | + | - | - | - | - | - |
| *atp6* | + | + | + | + | + | + | + | + | - | - | - |
| *atp8* | + | + | + | + | + | + | - | + | - | - | - |
| *atp9* | + | + | + | + | + | + | + | - | - | - | - |
| Large subunit ribosomal proteins | | | | | | | | | | | |
| *rpl5* | + | + | + | + | - | + | - | - | - | - | - |
| *rpl6* | + | + | + | + | - | - | - | - | - | - | - |
| *rpl14* | + | + | - | - | - | + | - | - | - | - | - |
| *rpl16* | + | + | + | + | + | + | - | - | - | - | - |
| Small subunit ribosomal proteins | | | | | | | | | | | |
| *rps2* | + | + | + | + | + | + | - | - | - | - | - |
| *rps3* | + | + | + | + | + | + | - | - | - | - | - |
| *rps4* | + | + | + | + | - | + | - | - | - | - | - |
| *rps7* | + | + | + | + | - | - | - | - | - | - | - |
| *rps8* | + | + | - | - | - | - | - | - | - | - | - |
| *rps10* | + | + | + | + | - | + | - | - | - | - | - |
| *rps11* | + | + | + | + | + | + | - | - | - | - | - |
| *rps12* | + | + | + | + | + | + | - | - | - | - | - |
| *rps13* | + | + | + | + | + | + | - | - | - | - | - |
| *rps14* | + | + | + | + | + | + | - | - | - | - | - |
| *rps19* | + | + | + | + | + | + | - | - | - | - | - |
| Sec-independent translocase | | | | | | | | | | | |
| *tatC* (*mttB*) | + | + | + | + | + | + | - | - | - | - | - |
| Ribosomal RNAs | | | | | | | | | | | |
| *rnl* | + | + | + | + | + | + | +^b^ | +^b^ | +^b^ | +^b^ | +^b^ |
| *rns* | + | + | + | + | + | + | +^b^ | + | +^b^ | +^b^ | +^b^ |
| *rrn5* | + | + | + | + | + | - | - | - | - | - | - |
| RNase P RNA | | | | | | | | | | | |
| *rnpB* | + | + | - | - | - | - | - | - | - | - | - |
| Transfer RNAs | 26 | 26 | 25 | 26 | 24 | 25 | 27 | 8 | 3 | 3 | 3 |
