# Supplementary material for: The Mitochondrial Genome of the Entomoparasitic Green Alga Helicosporidium
Source: PLoS One. 2010 Jan 29;5(1):e8954. doi: 10.1371/journal.pone.0008954 (PMC2813288; doi:10.1371/journal.pone.0008954)
Supplement: Table S2 — tRNA gene repertoires of Helicosporidium and other chlorophyte mtDNAs. a Nol, Nephroselmis olivacea; Ota, Ostreococcus tauri; Hsp, Helicosporidium sp.; Pwi, Prototheca wickerhamii; Ovi, Oltmannsiellopsis viridis; Pak, Pseudendoclonium akinetum; Sob, Scenedesmus obliquus; Pmi, Pedinomonas minor; Cre, Chlamydomonas reinhardtii; Ceu, Chlamydomonas eugametos; Cel, Chlorogonium elongatum. Presence/absence of a gene is denoted by +/−. b Me, elongator methionine; Mf, initiator methionine. c Genome specifies a single trnM(cau). (0.09 MB DOC) [file pone.0008954.s005.doc]

| Gene | Nol^a^ | Ota | Hsp | Pwi | Ovi | Pak | Sob | Pmi | Cre | Ceu | Cel |
| --- | --- | --- | --- | --- | --- | --- | --- | --- | --- | --- | --- |
| *trnA*(ugc) | + | + | + | + | + | + | + | - | - | - | - |
| *trnC*(gca) | + | + | + | + | + | + | + | + | - | - | - |
| *trnD*(guc) | + | + | + | + | + | + | + | - | - | - | - |
| *trnE*(uuc) | + | + | + | + | + | + | + | + | - | - | - |
| *trnF*(gaa) | + | + | + | + | + | + | + | + | - | - | - |
| *trnG*(gcc) | - | + | - | + | + | - | - | - | - | - | - |
| *trnG*(ucc) | + | + | + | + | + | + | + | - | - | - | - |
| *trnH*(gug) | + | + | + | + | + | + | + | + | - | - | - |
| *trnI*(cau) | + | - | + | + | + | + | - | - | - | - | - |
| *trnI*(gau) | + | + | + | + | - | + | + | - | - | - | - |
| *trnI*(uau) | - | - | - | - | - | - | + | - | - | - | - |
| *trnK*(uuu) | + | + | + | + | + | + | + | - | - | - | - |
| *trnL*(aag) | - | - | - | - | - | - | + | - | - | - | - |
| *trnL*(caa) | - | - | - | - | - | - | + | + | - | - | - |
| *trnL*(cag) | - | - | - | - | - | - | + | - | - | - | - |
| *trnL*(cua) | - | - | - | - | - | - | + | - | - | - | - |
| *trnL*(gag) | - | + | - | - | - | - | - | - | - | - | - |
| *trnL*(uaa) | + | + | + | + | + | + | - | - | - | - | - |
| *trnL*(uag) | + | + | + | + | + | + | - | - | - | - | - |
| *trnMe*(cau)^b^ | + | + | + | + | + | + | + | - | c | c | c |
| *trnMf*(cau)^b^ | + | + | + | + | + | + | + | - | c | c | c |
| *trnN*(guu) | + | + | + | + | + | + | + | - | - | - | - |
| *trnP*(ugg) | + | + | + | + | + | + | + | - | - | - | - |
| *trnQ*(uug) | + | + | + | + | + | + | + | + | + | + | + |
| *trnR*(acg) | + | + | + | + | + | - | + | - | - | - | - |
| *trnR*(ccu) | - | - | - | - | - | - | + | - | - | - | - |
| *trnR*(ucg) | + | - | - | - | - | + | - | - | - | - | - |
| *trnR*(ucu) | + | + | + | + | + | + | + | - | - | - | - |
| *trnS*(gcu) | + | + | + | + | + | + | + | - | - | - | - |
| *trnS*(gga) | - | - | - | - | - | - | + | - | - | - | - |
| *trnS*(uga) | + | + | + | + | + | + | - | - | - | - | - |
| *trnT*(ggu) | + | + | - | - | - | - | - | - | - | - | - |
| *trnT*(ugu) | - | - | + | + | - | + | - | - | - | - | - |
| *trnV*(uac) | + | + | + | + | + | + | + | - | - | - | - |
| *trnW*(cca) | + | + | + | + | + | + | + | - | + | + | + |
| *trnW*(uca) | - | - | - | - | - | - | - | + | - | - | - |
| *trnY*(gua) | + | + | + | + | + | + | + | + | - | - | - |
