# Supplementary material for: The Mitochondrial Genome of the Entomoparasitic Green Alga Helicosporidium
Source: PLoS One. 2010 Jan 29;5(1):e8954. doi: 10.1371/journal.pone.0008954 (PMC2813288; doi:10.1371/journal.pone.0008954)
Supplement: Table S3 — Codon usage in the 32 protein-encoding genes of Helicosporidium mtDNA. a Percentage of each amino acid specified by the specified codon. b Anticodon of Helicosporidium mtDNA-encoded tRNA recognizing the corresponding codon. The following tRNAs with an uracyl in the first position of their anticodon are assumed to decode all four members of the four-codon families: alanine, GCN; glycine, GGN; leucine, CUN; proline, CCN; serine, UCN; threonine, ACN; valine, GUN. c Amino acids are labelled by their one-letter IUPAC code. Termination codons are indicated by asterisks. d In chlorophytes mtDNAs, the gene coding for tRNAThr (ugu) has been found only within the mitochondrial genomes of Helicosporidium, Prototheca and Pseudendoclonium. e The initiator and elongator tRNAMet (cau) are encoded by different genes. (0.07 MB DOC) [file pone.0008954.s006.doc]

| Codon | %^a^ | ac^b^ | aa^c^ | Codon | % | ac | aa | Codon | % | ac | aa | Codon | % | ac | aa |
| --- | --- | --- | --- | --- | --- | --- | --- | --- | --- | --- | --- | --- | --- | --- | --- |
| GCA | 38 | ugc | A | UAA | 94 | — | * | CUA | 4 | uag | L | AGU | 29 | gcu | S |
| GCC | 2 | ugc | A | UAG | 6 | — | * | CUC | 0 | uag | L | UCA | 30 | uga | S |
| GCG | 4 | ugc | A | UGA | 0 | — | * | CUG | 0 | uag | L | UCC | 1 | uga | S |
| GCU | 56 | ugc | A | CAA | 94 | uug | Q | CUU | 10 | uag | L | UCG | 1 | uga | S |
| AGA | 49 | ucu | R | CAG | 6 | uug | Q | UUA | 84 | uaa | L | UCU | 35 | uga | S |
| AGG | 1 | ucu | R | GAA | 93 | uuc | E | UUG | 2 | uaa | L | ACA | 43 | ugu | T^d^ |
| CGA | 6 | gcu | R | GAG | 7 | uuc | E | AAA | 95 | uuu | K | ACC | 1 | ugu | T^d^ |
| CGC | 1 | gcu | R | GGA | 46 | ucc | G | AAG | 5 | uuu | K | ACG | 2 | ugu | T^d^ |
| CGG | 0 | gcu | R | GGC | 2 | ucc | G | AUG | 100 | cau | M^e^ | ACU | 54 | ugu | T^d^ |
| CGU | 43 | acg | R | GGG | 7 | ucc | G | UUC | 24 | gaa | F | UGG | 100 | cca | W |
| AAC | 7 | guu | N | GGU | 45 | ucc | G | UUU | 76 | gaa | F | UAC | 5 | gua | Y |
| AAU | 93 | guu | N | CAC | 9 | gug | H | CCA | 40 | ugg | P | UAU | 95 | gua | Y |
| GAC | 10 | guc | D | CAU | 91 | gug | H | CCC | 1 | ugg | P | GUA | 53 | uac | V |
| GAU | 90 | guc | D | AUA | 42 | gau | I | CCG | 4 | ugg | P | GUC | 3 | uac | V |
| UGC | 9 | gca | C | AUC | 8 | gau | I | CCU | 55 | ugg | P | GUG | 3 | uac | V |
| UCU | 91 | gca | C | AUU | 50 | gau | I | AGC | 4 | gcu | S | GUU | 41 | uac | V |
